# Supplementary material for: Real-Time Visualization of Cytosolic and Mitochondrial ATP Dynamics in Response to Metabolic Stress in Cultured Cells
Source: Cells. 2023 Feb 22;12(5):695. doi: 10.3390/cells12050695 (PMC10000496; doi:10.3390/cells12050695)
Supplement: Supplementary file 1 [file cells-12-00695-s001.zip › smacATPi Manuscript Figures RESUBMISSION 2 DW.pptx]

## Slide 1
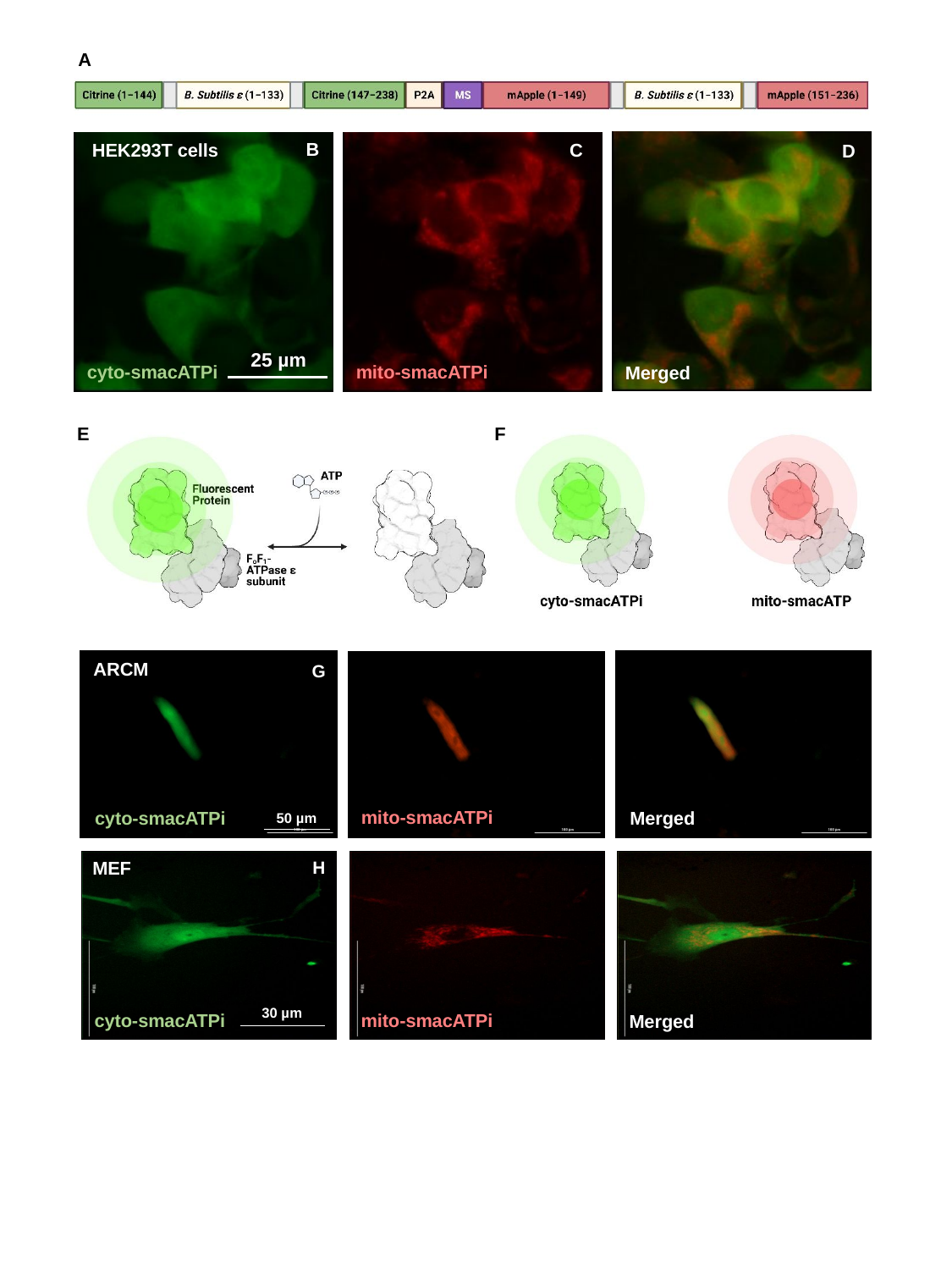

A
mito-smacATPi
cyto-smacATPi
Merged
25 µm
B
C
D
HEK293T cells
Cytosol
Mitochondria
E
F
ARCM
G
cyto-smacATPi
50 µm
mito-smacATPi
Merged
MEF
30 µm
mito-smacATPi
Merged
H
cyto-smacATPi

## Slide 2
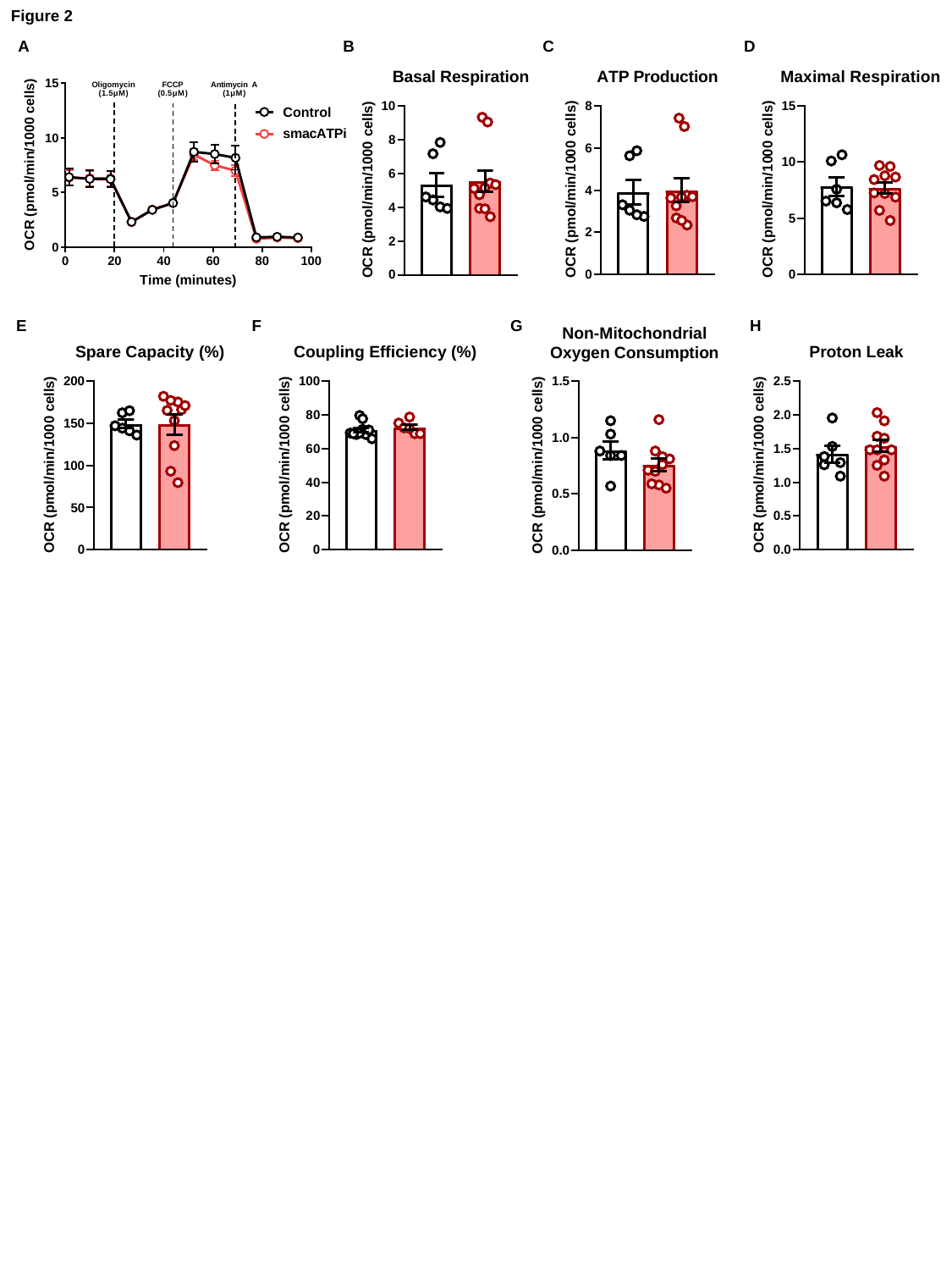

Figure 2
A
B
C
D
E
F
G
H

## Slide 3
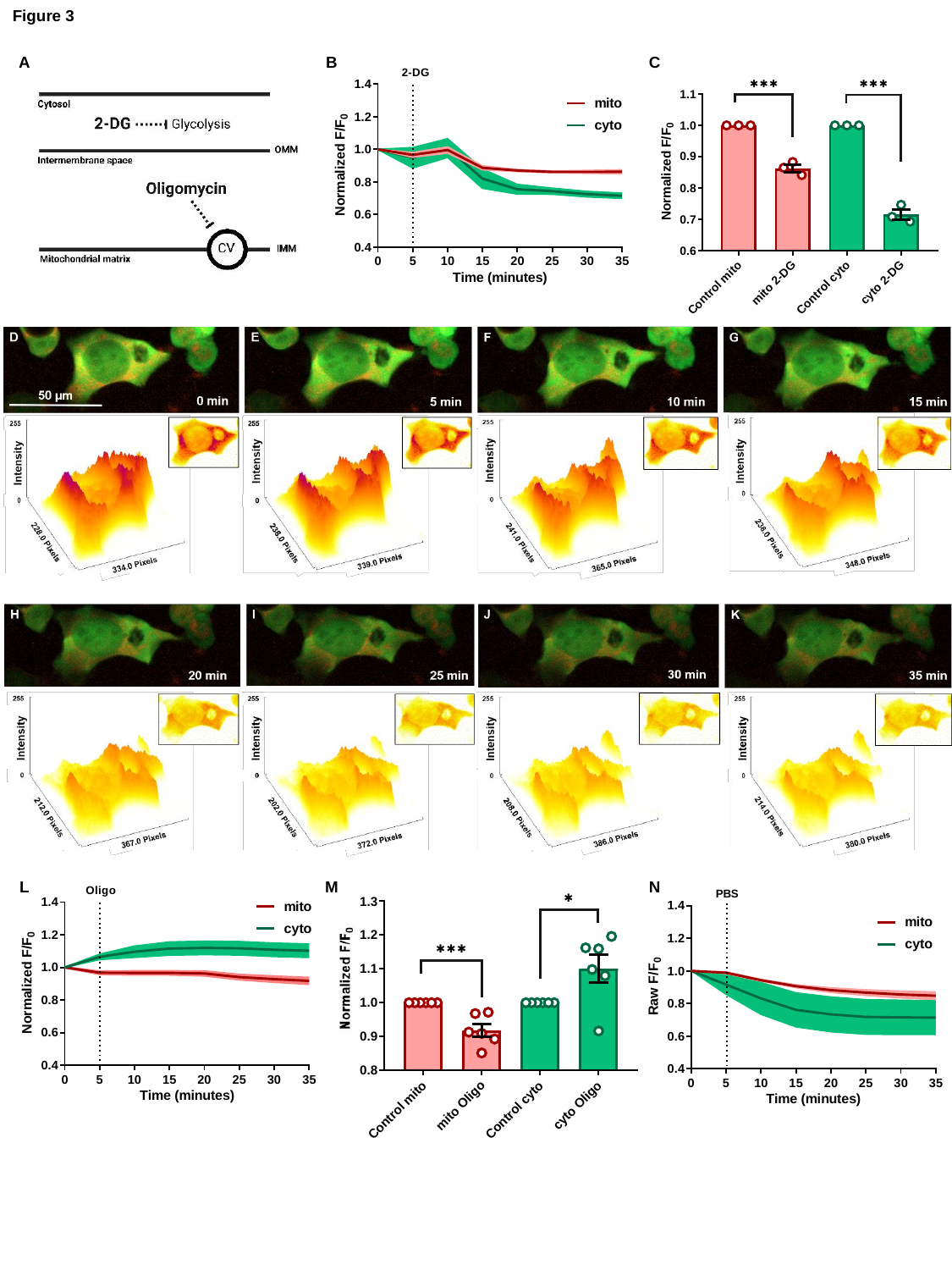

Figure 3
A
B
C
L
M
N

## Slide 4
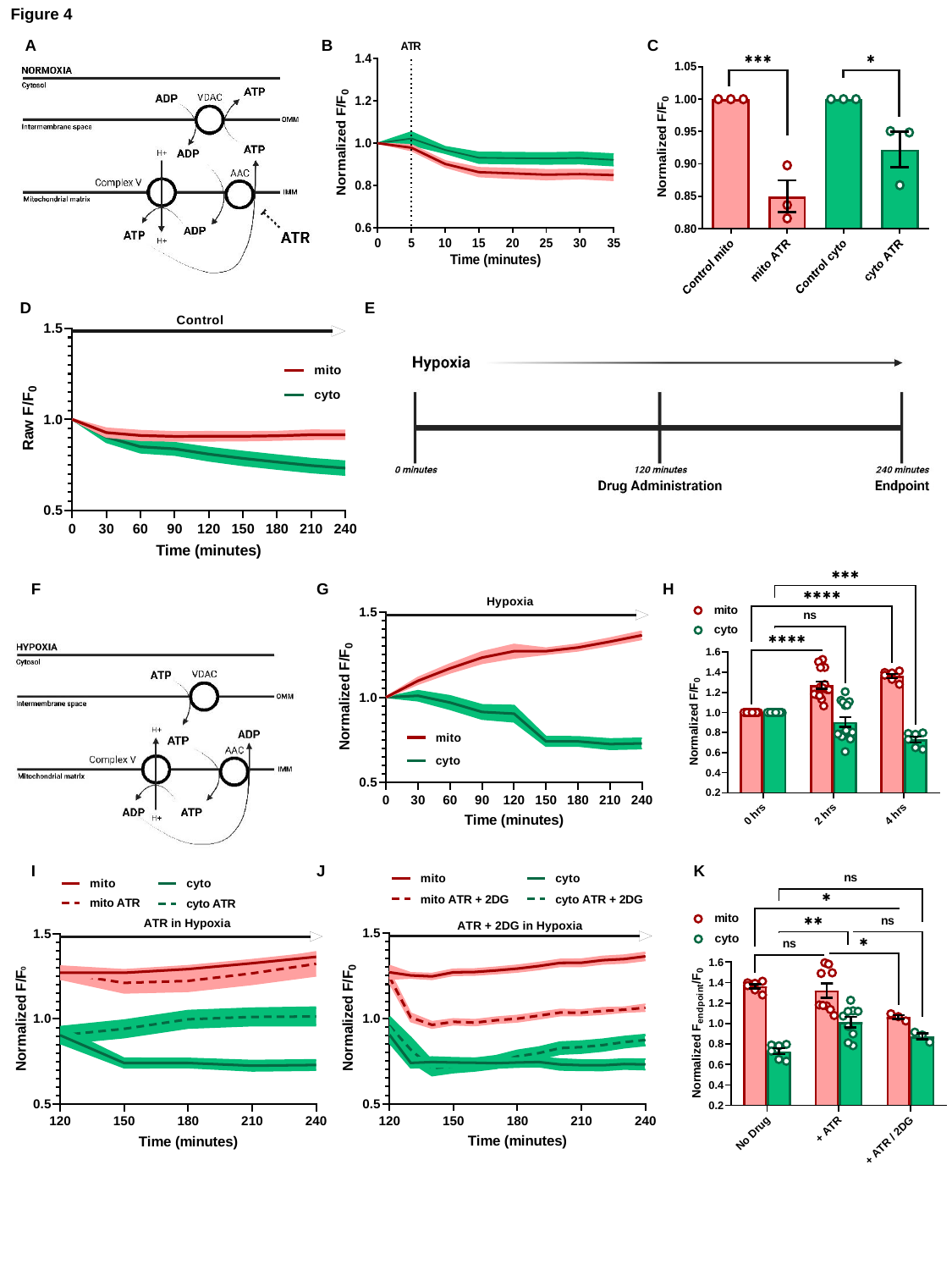

Figure 4
A
B
C
D
E
F
G
H
I
J
K

## Slide 5
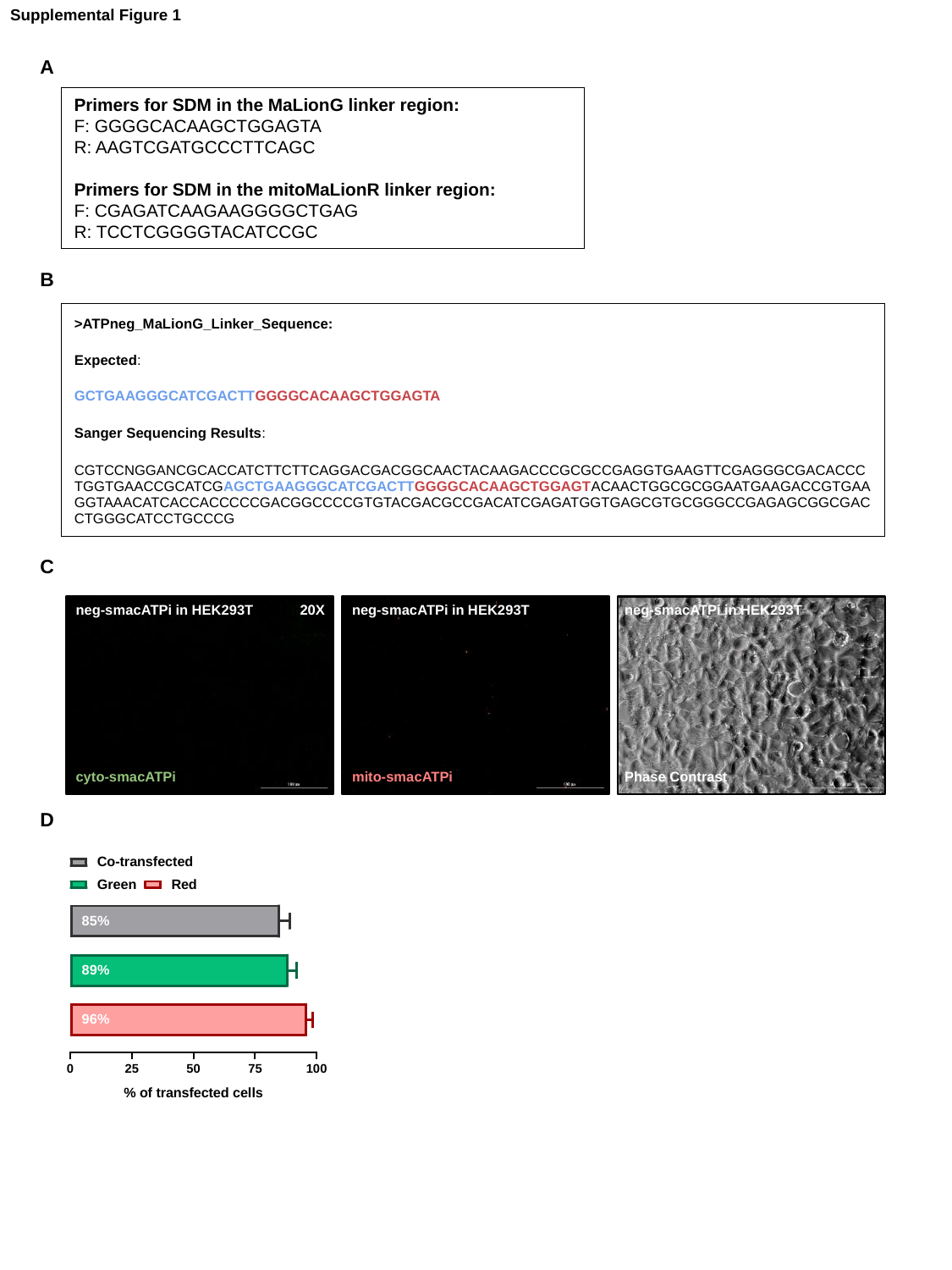

Supplemental Figure 1
A
Primers for SDM in the MaLionG linker region:
F: GGGGCACAAGCTGGAGTA
R: AAGTCGATGCCCTTCAGC
Primers for SDM in the mitoMaLionR linker region:
F: CGAGATCAAGAAGGGGCTGAG
R: TCCTCGGGGTACATCCGC
B
>ATPneg_MaLionG_Linker_Sequence:
Expected:
GCTGAAGGGCATCGACTTGGGGCACAAGCTGGAGTA
Sanger Sequencing Results:
CGTCCNGGANCGCACCATCTTCTTCAGGACGACGGCAACTACAAGACCCGCGCCGAGGTGAAGTTCGAGGGCGACACCCTGGTGAACCGCATCGAGCTGAAGGGCATCGACTTGGGGCACAAGCTGGAGTACAACTGGCGCGGAATGAAGACCGTGAAGGTAAACATCACCACCCCCGACGGCCCCGTGTACGACGCCGACATCGAGATGGTGAGCGTGCGGGCCGAGAGCGGCGACCTGGGCATCCTGCCCG
C
neg-smacATPi in HEK293T
cyto-smacATPi
neg-smacATPi in HEK293T
mito-smacATPi
20X
neg-smacATPi in HEK293T
Phase Contrast
D

## Slide 6
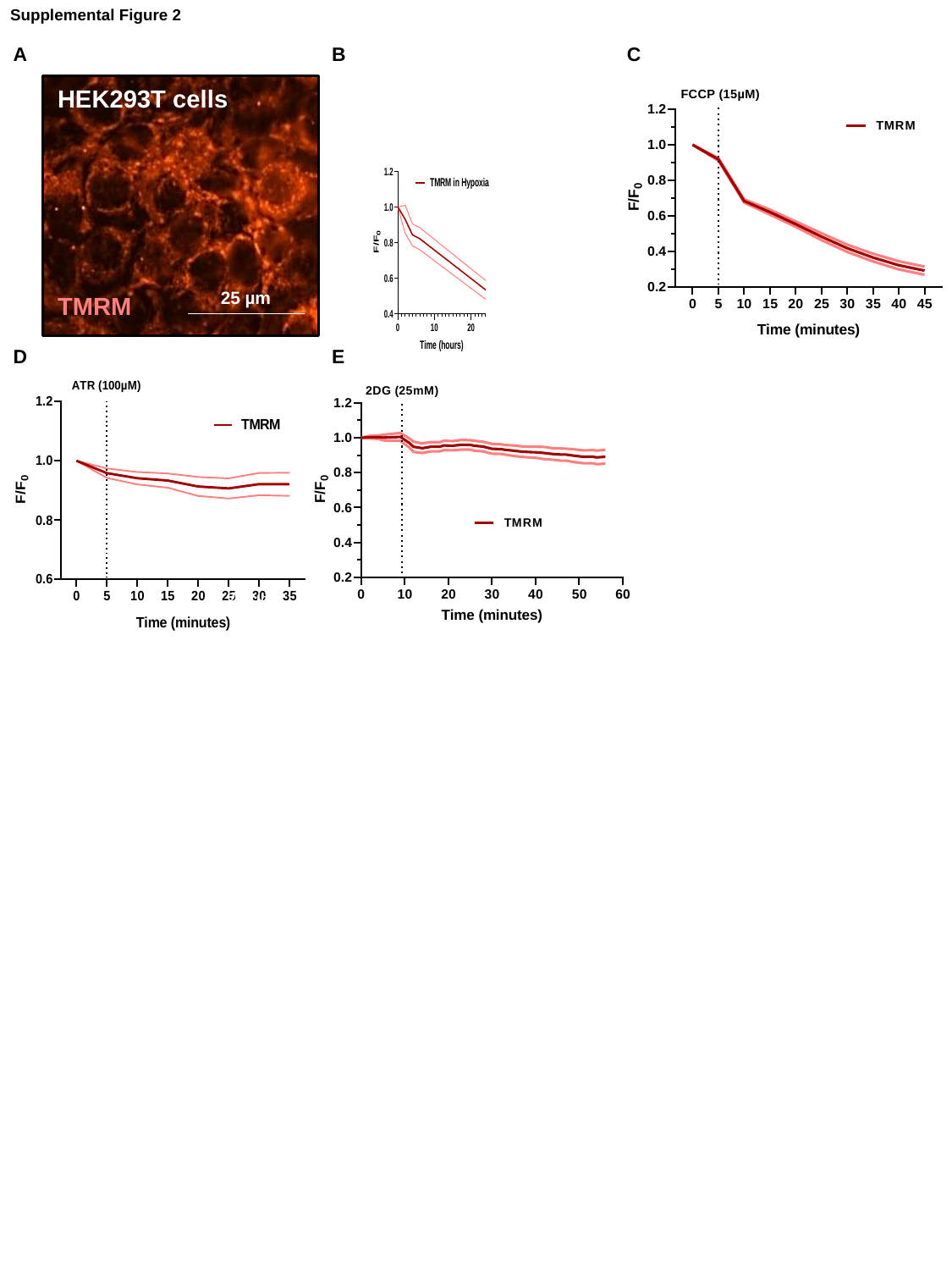

Supplemental Figure 2
A
B
C
HEK293T cells
TMRM
25 µm
D
E
25 µm

## Slide 7
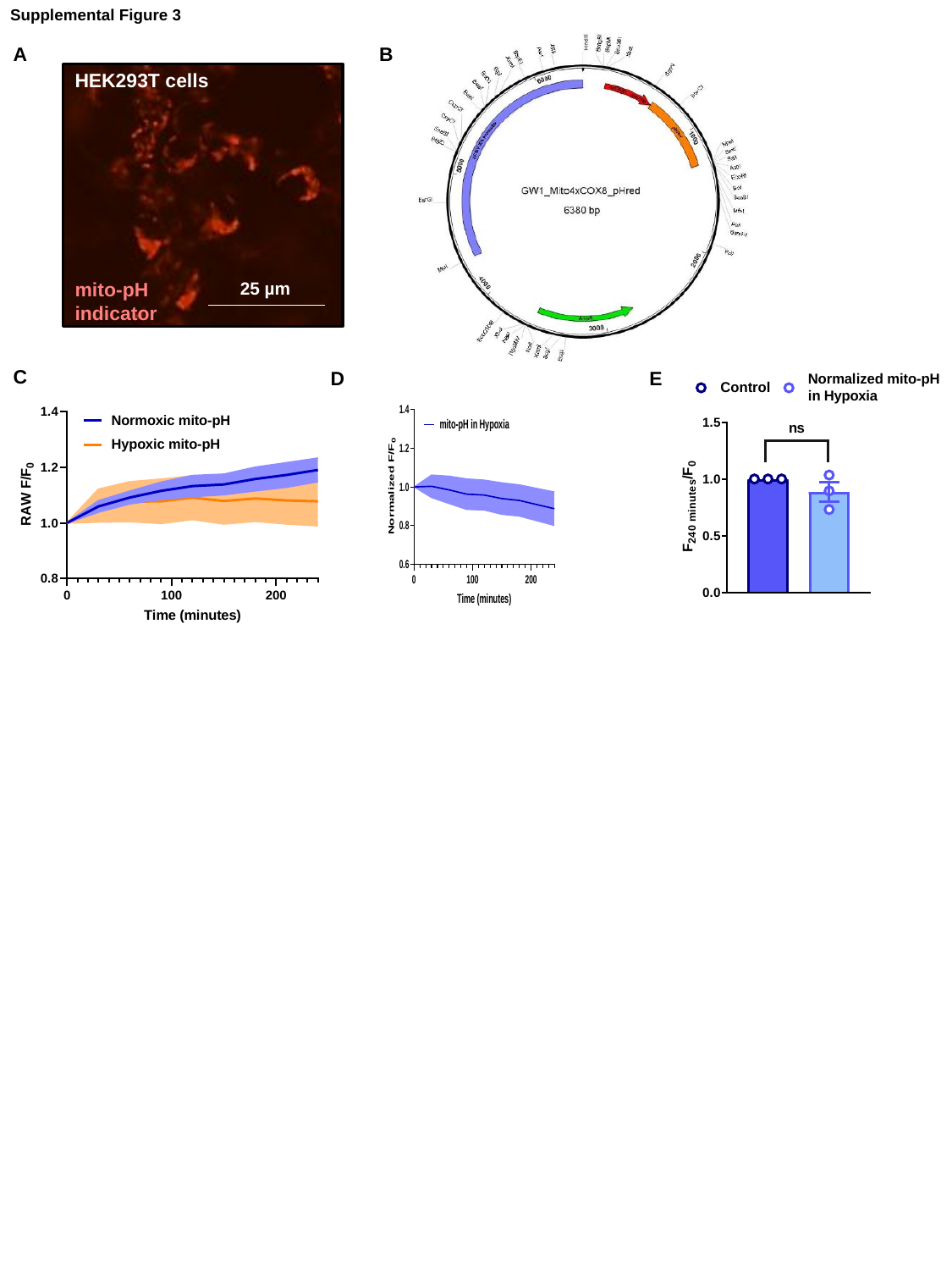

Supplemental Figure 3
B
A
HEK293T cells
mito-pH
indicator
25 µm
C
D
E

## Slide 8
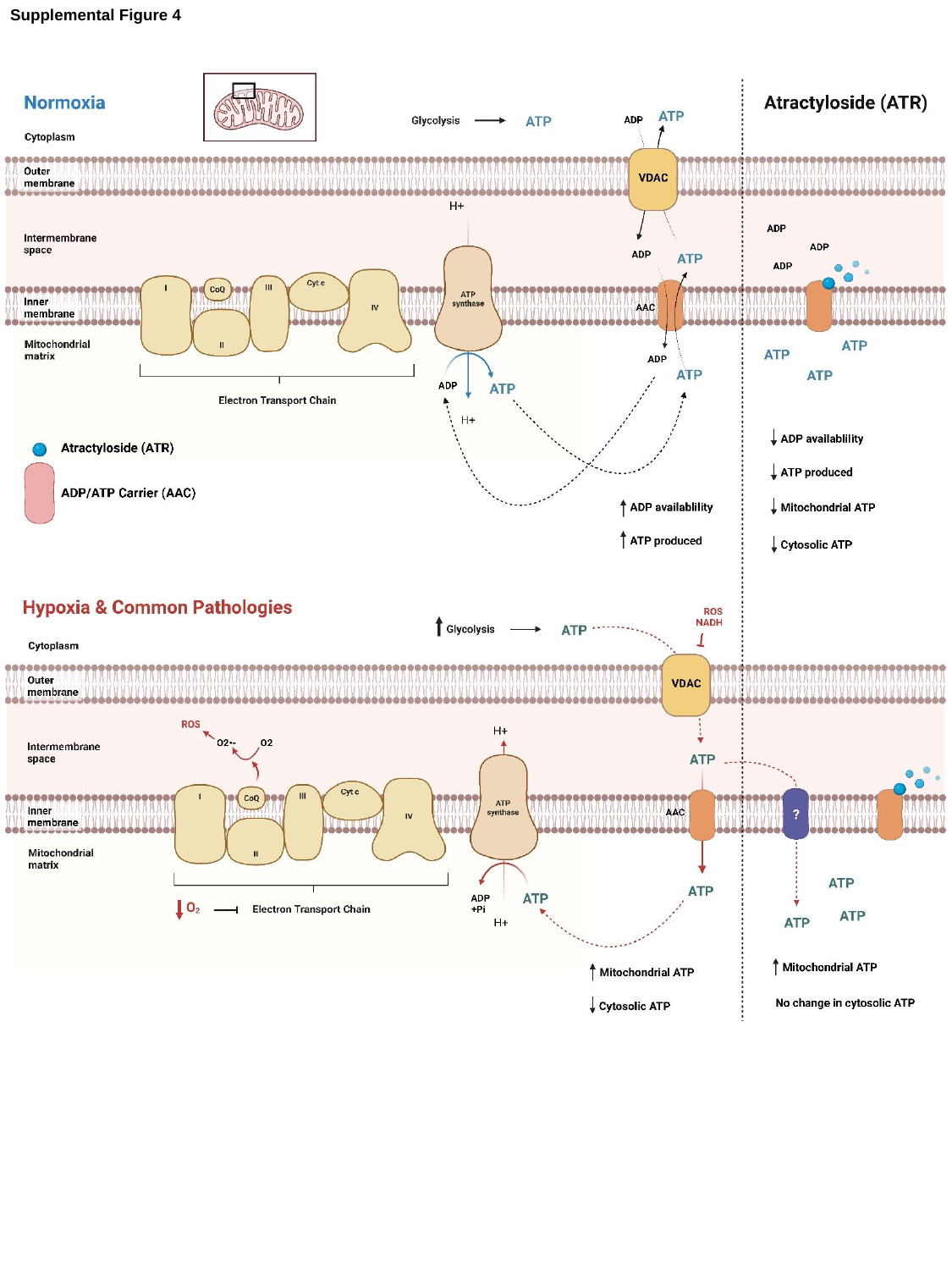

Supplemental Figure 4

## Slide 9
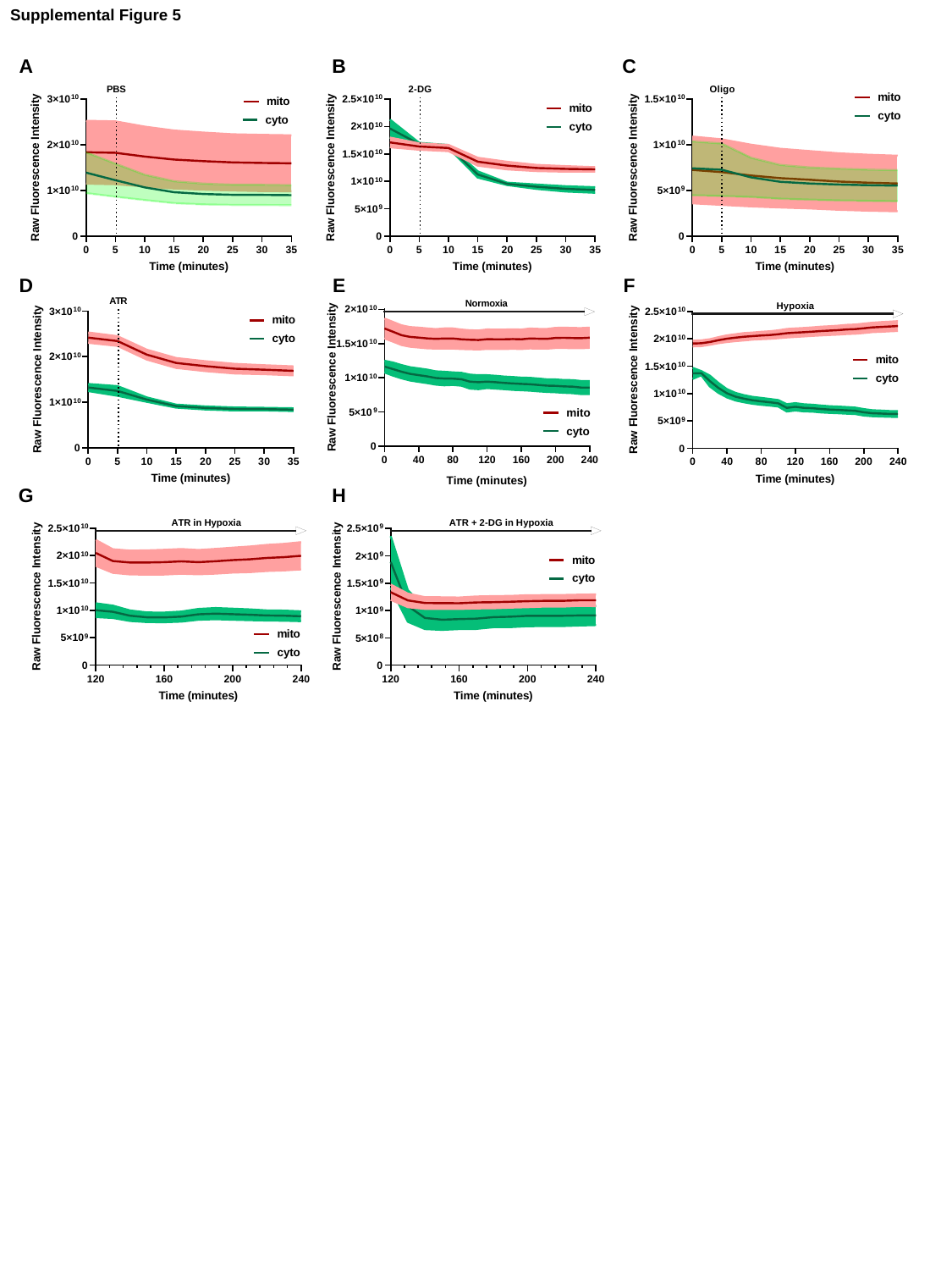

Supplemental Figure 5
A
B
C
D
E
F
G
H
