# Supplementary material for: Real-Time Visualization of Cytosolic and Mitochondrial ATP Dynamics in Response to Metabolic Stress in Cultured Cells
Source: Cells. 2023 Feb 22;12(5):695. doi: 10.3390/cells12050695 (PMC10000496; doi:10.3390/cells12050695)
Supplement: Supplementary file 1 [file cells-12-00695-s001.zip › smacATPi Supplemental Figures RESUBMISSION 2 DW.pdf]

## Supporting Information for

# Real-time Visualization of Cytosolic and Mitochondrial ATP Dynamics in Response to Metabolic Stress in Cultured Cells

**Donnell White, III[1,2,3,] Lothar Lauterboeck[1,2,] Parnia Mobasheran[1,2,] Tetsuya Kitaguchi[4], Antoine H. Chaanine[5], and Qinglin Yang*[1,2]**

1 Cardiovascular Center of Excellence, Louisiana State University Health Sciences Center, New Orleans, LA 70112, USA
2 Department of Pharmacology and Experimental Therapeutics, School of Graduate Studies, Louisiana State University Health Sciences Center, New Orleans, LA 70112, USA
3 School of Medicine, Louisiana State University Health Sciences Center, New Orleans, LA 70112, USA
4 Cell Biology, Life Science Solutions, Thermo Fisher Scientific, Frederick, MD 21702, USA
5 Laboratory for Chemistry and Life Science, Institute of Innovative Research, Tokyo Institute of Technology, 4259 Nagatsuta-cho, Midori-ku, Yokohama, Kanagawa 226-8503, Japan
6 Regions Hospital, HealthPartners Group, Saint Paul, MN 55101, USA

*Corresponding to:

**Email**: qyang1@lsuhsc.edu

**This PDF file includes:**

Figures S1 to S5
Legends for Movies S1 to Sx

**Other supporting materials for this manuscript include the following:**

Movies S1 to Sx
Datasets S1 to Sx
Software S1 to Sx

Supplemental Figure 1

**A**

**Primers for SDM in the MaLionG linker region:**
F: GGGGCACAAGCTGGAGTA
R: AAGTCGATGCCCTTCAGC

**Primers for SDM in the mitoMaLionR linker region:**
F: CGAGATCAAGAAGGGGCTGAG
R: TCCTCGGGGTACATCCGC

**B**

**>ATPneg_MaLionG_Linker_Sequence:**

**Expected**:

**GCTGAAGGGCATCGACTTGGGGCACAAGCTGGAGTA**

**Sanger Sequencing Results**:

CGTCCNGGANCGCACCATCTTCTTCAGGACGACGGCAACTACAAGACCCGCGCCGAGGTGAAGTTCGAGGGCGACACCC
TGGTGAACCGCATCG**AGCTGAAGGGCATCGACTTGGGGCACAAGCTGGAGT**ACAACTGGCGCGGAATGAAGACCGTGA
AGGTAAACATCACCACCCCCGACGGCCCCGTGTACGACGCCGACATCGAGATGGTGAGCGTGCGGGCCGAGAGCGGCG
ACCTGGGCATCCTGCCCG

**C**

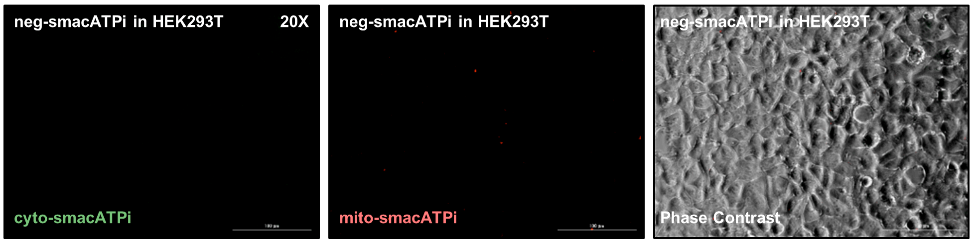


**D**

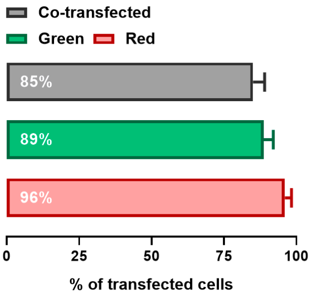


**Figure S1. A mutated form of smacATPi (neg-smacATPi), where small deletions were introduced into the linker regions of the fluorescent proteins to exclude the possibility that the fluorescent signals are derived from autofluorescence. The rate of efficiency for co-transfection of both**

**smacATPi indicators (mito and cyto) is high.** A) The specific primers used for mutagenesis are shown. B) The cyto-smacATPi deletion was verified via sanger sequencing. C) Transfection of the mutated plasmid containing small deletions in the linker regions of both the cyto-smacATPi and mito-smacATPi showed no fluorescence. D) Although smacATPi codes for two individual compartmental indicators, there is very high co-expression of both indicators in transfected HEK293T cells (n=3 fields, with each field containing >25 cells).

Supplemental Figure 2

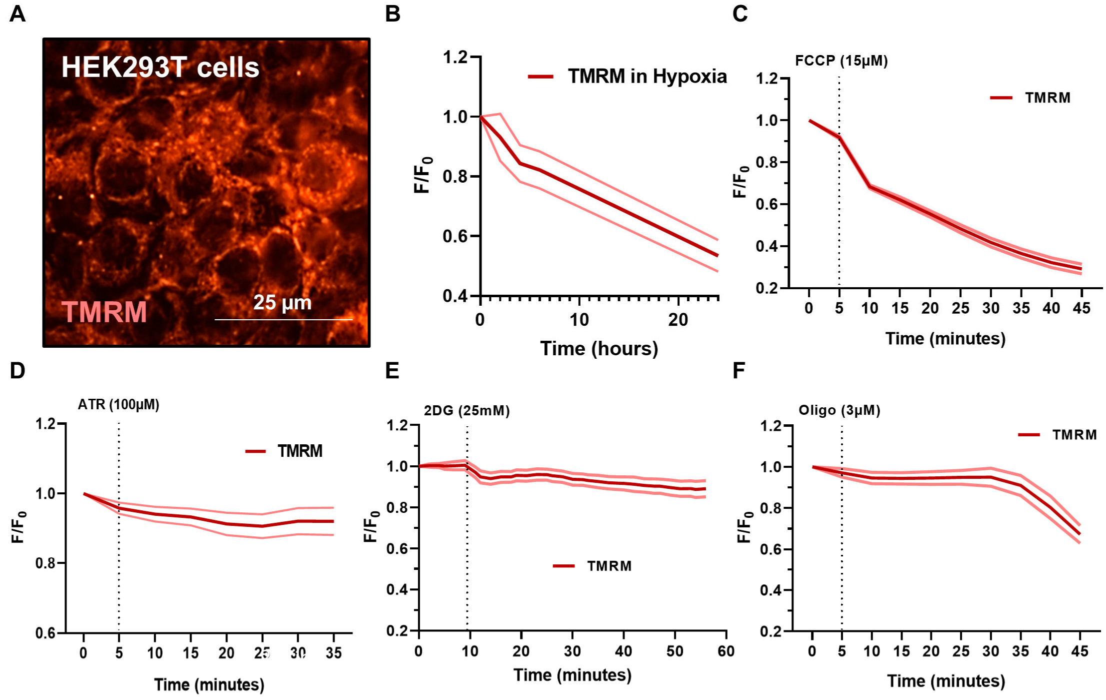


**Figure S2. Mitochondrial membrane potential assays in HEK293T cells subjected to hypoxia.** A) A representative image of HEK293T cells with TMRM staining. B) TMRM assay of mitochondrial membrane potential in cultured HEK293T cells subjected to 4-hour hypoxia (2% $O_2$). C) FCCP (15μM) treatment on HEK293T cells under normoxia shows an expected decrease in mitochondrial membrane potential, confirming that TMRM is working effectively. D) ATR (100μM) was administered to TMRM-stained HEK293T cells, and there was little depolarization in this case. E) 2-DG (25mM) was also given to these cells. (n=3 or more fields, with each field containing >10 cells; error bars are SEM; ns P>0.05, * P≤0.05, ** P≤0.01, *** P≤0.001, **** P≤0.0001).

Supplemental Figure 3

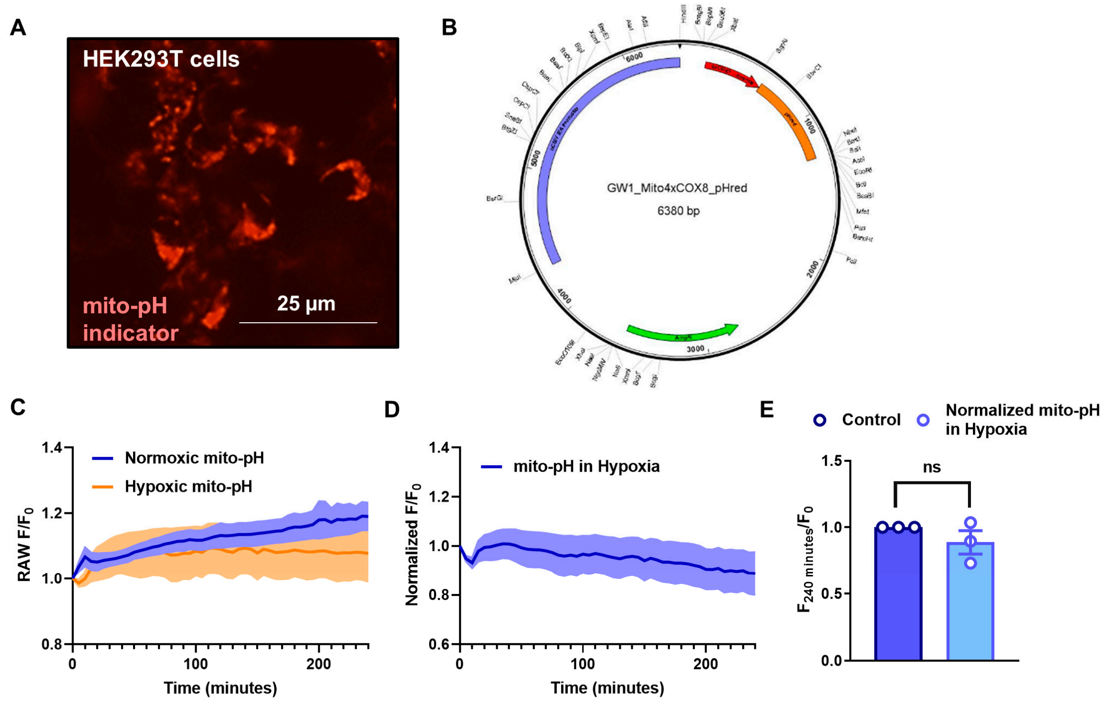


**Figure S3. Mitochondrial pH changes during hypoxia.** A) A mito-pH indicator was used to determine whether pH fluctuations interfere with the fluorescent signals of smacATPi. B) The pH indicator used was GW1-Mito-pHRed (Addgene plasmid #31474). C) Raw fluorescent intensity from HEK293T cells transfected with mito-pH tracker over a 4-hour period under normoxia and hypoxia. D) The fluorescent intensity in hypoxia normalized to normoxia. E) The fluorescent intensity in response to pH change in hypoxic conditions. (n=3 or more fields, with each field containing >10 cells; error bars are SEM; ns P>0.05, * P≤0.05, ** P≤0.01, *** P≤0.001, **** P≤0.0001).

Supplemental Figure 4

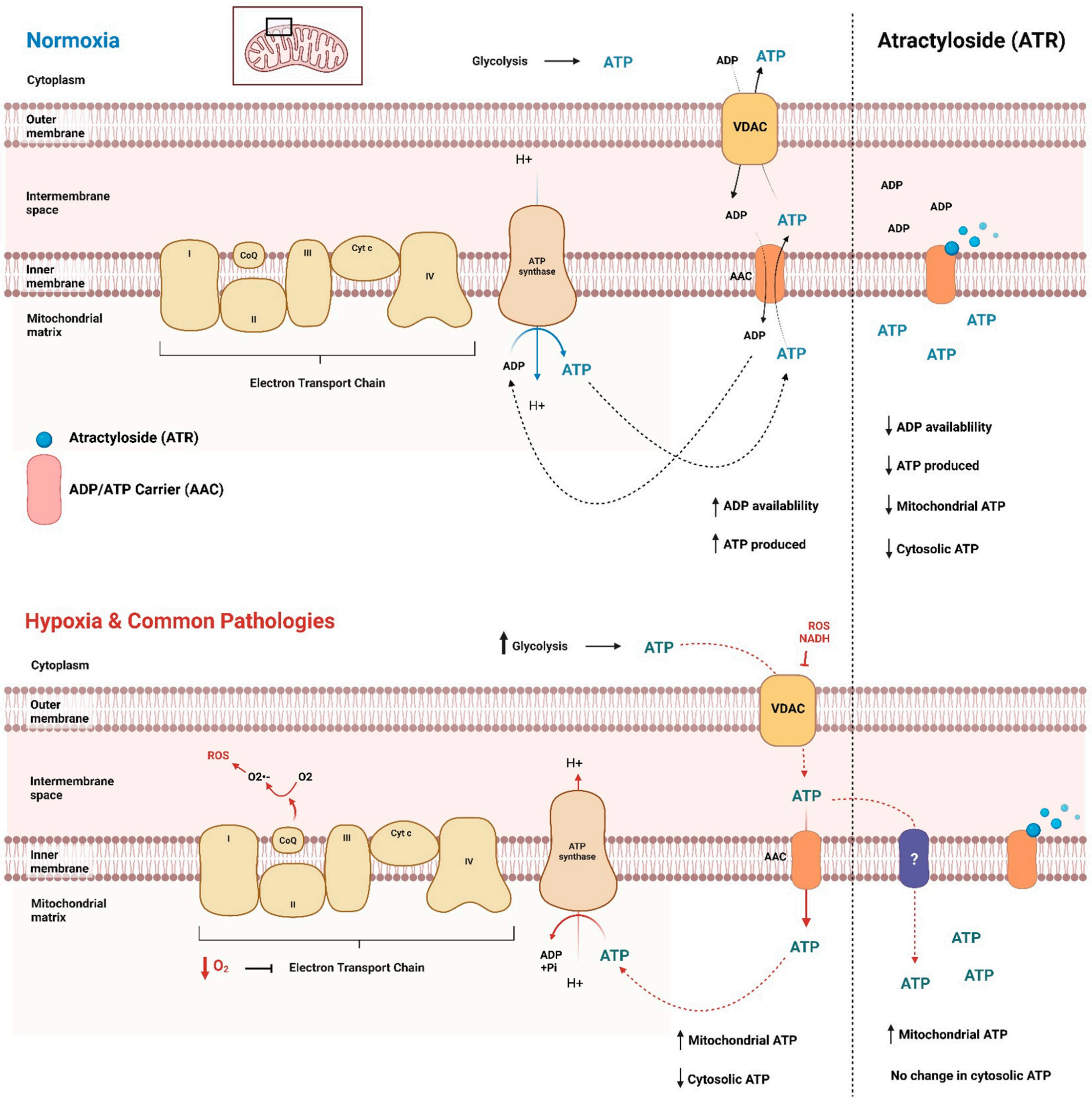


**Figure S4. ATP movement between mitochondria and cytosol under normoxic and hypoxic conditions in the presence of ATR.** Under normoxic conditions, the cell transports ATP both into and out of the mitochondrial matrix. Mitochondria produces most ATP in normoxic conditions (~90%), while glycolysis produces a significantly lower amount (~10%). However, hypoxia results in the

reversal of ATP transport to inside the matrix via AAC resulting in increased mitochondrial ATP, while there is a decrease in cytosolic ATP availability. When atractyloside (ATR) is given to these cells under normoxic conditions, we see a decrease in both mitochondrial and cytosolic ATP. When cells are pre-conditioned to hypoxic conditions before ATR is given, mitochondrial ATP was increased as previously seen under hypoxic conditions. The unchanged cytosolic ATP, suggesting that an uncharacterized rescue mechanism allows ATP to be transported into the matrix.

Supplemental Figure 5

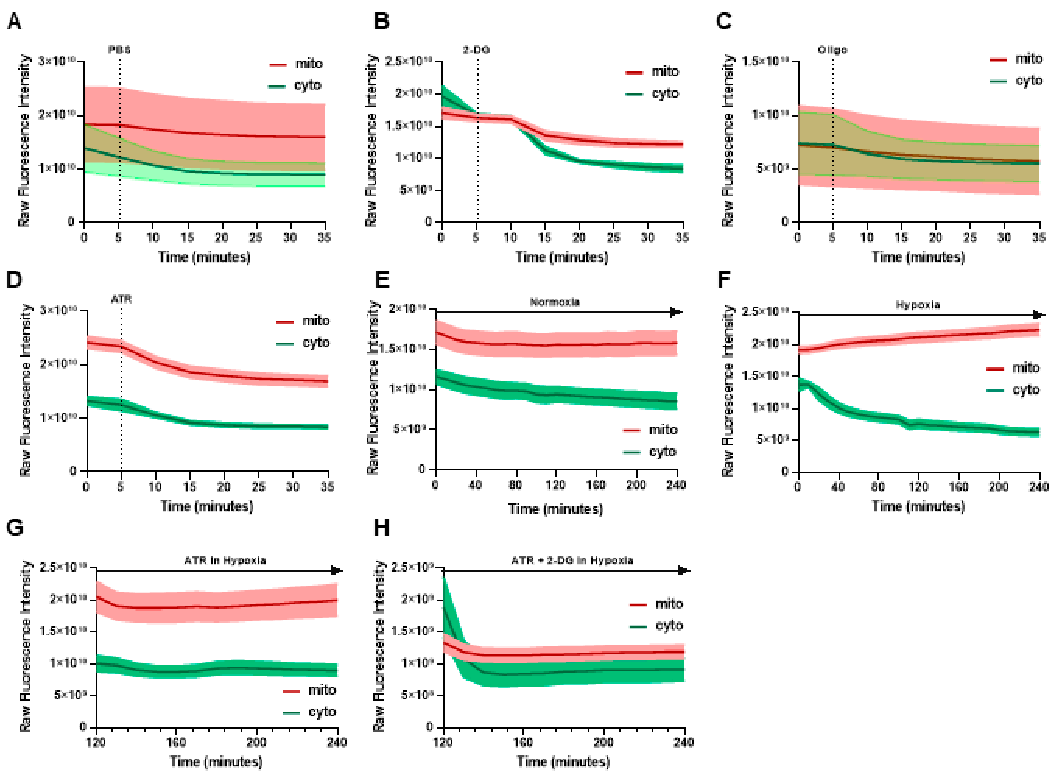


**Figure S5. Raw fluorescent intensities from Cytation5.** A) Raw fluorescent intensity from PBS administration for drug normalization. B) Raw fluorescent intensity from 2-DG administration. C) Raw fluorescent intensity from oligo administration. D) Raw fluorescent intensity from ATR administration. E) Raw fluorescent intensity from 4 hour normoxic control for hypoxia normalization. F) Raw fluorescent intensity from 4 hour hypoxia. G) Raw fluorescent intensity from ATR administration in hypoxia. H) Raw fluorescent intensity from ATR and 2-DG administration in hypoxia. (n=3 or more fields, with each field containing >10 cells; error bars are SEM; ns P>0.05, * P≤0.05, ** P≤0.01, *** P≤0.001, **** P≤0.0001).

**Movie S1 (separate file). A vehicle control (PBS) was added to cells for normalization of drug trials.**

**Movie S2 (separate file). 2DG was added to smacATPi-expressing HEK293T cells.**

**Movie S3 (separate file). smacATPi-expressing HEK293T cells were treated with Oligomycin.**

**Movie S4 (separate file). ATR was added to smacATPi-expressing HEK293T cells.**

**Movie S5 (separate file). A control 4-hour run was done for normalization of hypoxic conditions.**

**Movie S6 (separate file). smacATPi-expressing HEK293T cells were subjected to 4-hours of hypoxic conditions (5% $CO_2$ and 2% $O_2$).**

**Movie S7 (separate file). ATR was added to smacATPi-expressing HEK293T cells 2 hours post-exposure to hypoxic conditions (5% $CO_2$ and 2% $O_2$).**
